# Electrochemical Evaluation of New Ti-Based High-Entropy Alloys in Artificial Saliva with Fluoride: Implications for Dental Implant Applications

**DOI:** 10.3390/ma18132973

**Published:** 2025-06-23

**Authors:** Hanine Slama, Qanita Tayyaba, Mariya Kadiri, Hendra Hermawan

**Affiliations:** Department of Mining, Metallurgical and Materials Engineering, Laval University, Quebec City, QC G1V 0A6, Canada; hanine.slama.1@ulaval.ca (H.S.); qanita.tayyaba.1@ulaval.ca (Q.T.); mariya.kadiri.1@ulaval.ca (M.K.)

**Keywords:** artificial saliva, biomaterials, dentistry, fluoride-induced corrosion, high-entropy alloys, titanium

## Abstract

Based on their high mechanical strength, Ti-based high-entropy alloys (HEAs) are of great potential as materials for high-performance reduced-diameter dental implants. Despite previous studies demonstrating their corrosion resistance in various simulated body fluids, their resistance in simulated buccal conditions has yet to be confirmed. In this work, the corrosion behavior of two Ti-based HEAs, TiZrHfNb, and TiZrHfNbTa was evaluated in comparison to CP-Ti and Ti-6Al-4V in artificial saliva (AS) solution and in AS with fluoride ion content (ASF). A set of electrochemical tests (electrochemical impedance spectroscopy, cyclic polarization, and Mott–Schottky) was employed and complemented with surface characterization analyses (scanning electron microscopy and atomic force microscopy) to determine dissolution and passivation mechanisms of the alloys. In general, the HEAs exhibited a far superior corrosion resistance compared to CP-Ti and Ti-6Al-4V alloys in both solutions. In the AS solution, the TiZrHfNb exhibited the highest polarization resistance and pitting potential, indicating a high corrosion resistance due to the formation of a robust passive layer. Whilst in the ASF solution, the TiZrHfNbTa showed a greater corrosion resistance due to the synergistic effect of Nb and Ta oxides that enhanced passive film stability. This finding emphasizes the role of Ta in elevating the corrosion resistance of Ti-based HEAs in the presence of fluoride ions and confirms the importance of chemical composition optimization in the development of next-generation dental alloys. Based on its electrochemical corrosion behavior, TiZrHfNbTa HEAs are promising new materials for high-performance reduced-diameter dental implants.

## 1. Introduction

In contemporary dental implantology, the clinical need for reduced-diameter implants (RDIs) arises to facilitate thin alveolar ridges and minimize invasive treatments, primarily in older or medically compromised patients [1]. RDIs aim to decrease surgical trauma and healing durations with maintained functional and esthetic outcomes. However, RDIs require high mechanical stress, and hence, there is a necessity for higher strength and resistance to fatigue on the implant materials’ side. Commercially pure titanium (CP-Ti) and Ti alloys, especially Ti-6Al-4V and Ti-Zr, are the current gold standards due to their biocompatibility, osseointegration, and resistance to corrosion [2,3]. However, their mechanical limitation, particularly in terms of the smaller cross-sectional area, is a question of fatigue failure and implant fracture [4]. Some reports suggest that even Ti-Zr alloys, despite being stronger than CP-Ti, may not be strong enough for the long-term success of RDIs [5]. These limitations emphasize the imperative need for novel high-performance materials that are mechanically sound and biocompatible.

High-entropy alloys (HEAs) are a newly discovered family of biomedical materials that also comprise dental implants. Characterized by the multi-principal element composition, HEAs exhibit unique microstructures accountable for exceptional mechanical properties such as incredibly high tensile strength, remarkable fatigue resistance, and elevated hardness that outshines that of conventional Ti-alloys such as Ti-6Al-4V and Ti-Zr under equal loading conditions [6,7]. Interestingly, Ti-based HEAs (e.g., TiNbZrTaMo and TiTaHfNbZr) possess Young’s modulus values closer to that of bone and offer increased fracture toughness, both being vital in reduced-diameter applications [8,9]. Furthermore, HEAs have also been observed to possess greater corrosion resistance in simulated body fluid, i.e., Hanks’ solution, which is equivalent to or superior to CP-Ti and Ti-alloys due to the existence of stable, passive Cr, Ti, and Ta-rich oxide films [10,11,12]. Yet few research works have investigated the corrosion behavior of HEAs under simulated buccal conditions, using artificial saliva solution, a chemical test for its intricate ionic make-up and dynamic pH [13]. It is among the key gaps in the existing knowledge that the present work aims to fill.

The oral cavity imposes a particularly harsh environment on metallic biomaterials in the guise of its dynamic chemical environment, recurrent exposure to fluoride-containing products, and biofilm growth. Artificial saliva solution, used to simulate the environment, is generally made up of additives like NaCl, KCl, CaCl_2_, urea, and mucin. Fluoride, which occurs in oral hygiene products, can destabilize passive films on Ti and Ti-alloys and accelerate pitting and crevice corrosion, especially at low pH [14,15,16]. Several studies have completely explained the degradation mechanisms of Ti-6Al-4V and Ti-Zr in the AS and its variants, comprising fluoride-induced surface roughening, ion release, and microstructure damage [17]. These findings emphasize the need to test new materials under conditions that are most like the buccal environment. Since HEAs have shown superior performance in other simulated body fluids and their desirable mechanical profile, we expect that Ti-based HEAs would exhibit corrosion resistance in the artificial saliva solution equal to or superior to Ti-alloys [18]. Therefore, this work aims to assess the electrochemical corrosion behavior of selected Ti-based HEAs under simulated oral conditions to verify their potential use for reduced-diameter dental implants.

By combining a set of electrochemical tests (open circuit potential, electrochemical impedance spectroscopy, potentiodynamic polarization, and Mott–Schottky) in the artificial saliva solution, this work will reveal the general corrosion characteristics of two Ti-based HEAs (TiZrHfNb and TiZrHfNbTa) in comparison to CP-Ti and Ti-6Al-4V. Further analysis by means of potentiostatic polarization, scanning electron microscope (SEM), energy dispersive X-ray spectrometer (EDX), and atomic force microscope (AFM) should reveal the underlying mechanism of passivation on the two HEAs in the simulated buccal conditions. The gained understanding will contribute to the advancement of knowledge of HEAs for biomedical applications and be the foundation of future-generation dental implant design with reduced-diameter and high-performance clinical uses in mind.

## 2. Materials and Methods

### 2.1. Alloy Preparation

Two as cast HEAs samples, TiZrHfNb and TiZrHfNbTa, were prepared using high-purity elemental metals (≥99.9%) of titanium (Ti), zirconium (Zr), hafnium (Hf), niobium (Nb), and tantalum (Ta) through an arc melting process, as detailed in a previous work [19]. The TiZrHfNb was designed to have a composition of Ti (29.41 at. %), Zr (29.41 at. %), Hf (29.41 at. %), and Nb (11.76 at. %), while the TiZrHfNbTa was to have all five elements in the same atomic percents (20 at. % each) as shown in Table 1. The addition of Ta was aimed at enhancing the stability of oxide films [20]. Commercially pure Ti (CP-Ti) and Ti-6Al-4V samples were also used for comparison purposes (McMaster-Carr, Cleaveland, OH, USA). All samples were sectioned, mechanically ground, and polished to a mirror finish for subsequent electrochemical and surface characterization.

### 2.2. Electrochemical Testing

The electrochemical testing was performed with a three-electrode arrangement using a potentiostat (Interface 1010E, Gamry Instruments, Warminster, PA, USA) where the metal samples, saturated calomel electrode (SCE), and graphite rod served as working, reference, and counter electrodes, respectively. The tests were conducted at 37 ± 1 °C in an artificial saliva (AS) solution and in AS with fluoride ion content (ASF). The AS solution was prepared with analytical-grade reagents (Sigma-Aldrich Canada, Oakville, ON, Canada) by dissolving the following in 1 L of distilled water as shown in Table 2. To formulate the ASF, NaF was added at a concentration of 12.3 g/L, which is equivalent to 22,600 ppm of fluoride ions [21,22].

The metal samples were stabilized at their open circuit potential (OCP) for 1 h before being subjected to the electrochemical tests. Electrochemical impedance spectroscopy (EIS) analysis was performed to reveal interfacial properties by applying a 10 mV square root mean sinusoidal perturbation over a frequency range from 100 kHz to 10 mHz. The obtained Nyquist plots were fitted with equivalent circuit models (ECM). Cyclic potentiodynamic polarization (CPP) tests were performed to determine corrosion behavior by using a scan rate of 1 mV/s, and Tafel extrapolation to obtain corrosion parameters. Mott–Schottky tests were performed at a potential range of −2.0 V to +2.0 V with an increment of 0.1 V to examine the semiconducting nature of the passive film. CP-Ti and Ti-6Al-4V were also electrochemically characterized for comparison. Additionally, potentiostatic polarization (PSP) tests were performed on the HEA samples for 1 h at a pitting potential derived from the obtained CPP plots to assess passive film stability. Each experiment was performed in triplicate to ensure reproducibility. Data acquisition and analysis were carried out using Gamry Echem Analyst software version 7.10.4.14736 (Gamry Instruments, Warminster, PA, USA) and Origin software version 2024b (OriginLab Corporation, Northampton, MA, USA), respectively.

### 2.3. Surface Characterization

The general microstructure and surface morphology of the samples after being subjected to the PSP tests were observed using an SEM/EDX (Inspect F50, FEI, Hillsboro, OR, USA). Characterization of the passive film of CP-Ti and all HEA samples was performed before and after the PSP tests and the change in the surface topography as well as the roughness at scan size of 120 μm^2^ was measured using an AFM (MFP-3D Origin+, Oxford Instruments, Santa Barbara, CA, USA) in a contact mode in air.

## 3. Results

### 3.1. Microstructural Observation

**Figure 1:**
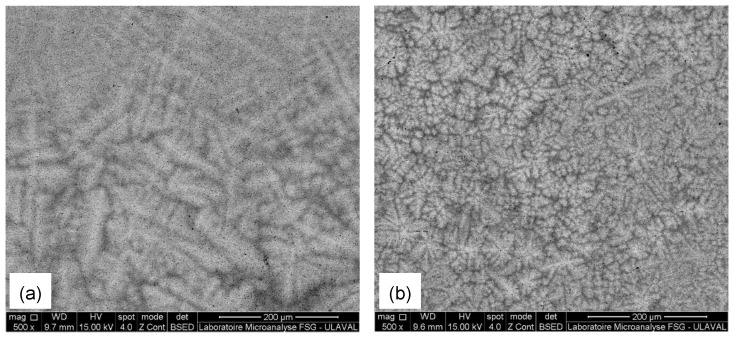
SEM micrographs of (**a**) TiZrHfNb and (**b**) TiZrHfNbTa.

The SEM microstructure of the TiZrHfNb alloy shown in Figure 1a reveals a compact and relatively homogenous morphology. The backscattered electron (BSE) image reveals the differences in contrast because of the elemental distribution, where the bright regions presumably are Hf-rich regions because of their higher atomic number, and the darker regions are for Ti-dominant regions. The morphology shows the presence of fine, evenly distributed dark streaks and isolated microstructural features, which are likely associated with Nb-rich regions or potential micro-segregation domains. No large precipitates, cracks, or porosity is present, testifying to excellent metallurgical quality and homogeneity within the solid solution matrix. In addition, with regard to the solidification behavior of TiZrHfNb composition in the literature, the formation of dendritic microstructures is common due to elemental segregation during solidification [23]. The current TiZrHfNb sample is not necessarily composed of distinct dendritic features on the observed length scales, but the total fine contrast variations may be suggestive of some compositional segregation [24].

The SEM microstructure of TiZrHfNbTa in Figure 1b is characterized by a multi-phase microstructure of inter-dendritic and dendritic phases reflecting compositional segregation through cooling rate variation during solidification. BSE image shows non-uniform distribution of phases with bright and dark contrast areas reflecting elemental partitioning. The morphology shows a flower-like morphology, likely caused by phase separation or localized element redistribution during solidification [25]. The general tendency for segregation of HEAs with the higher melting point elements, Ta (3290 K) and Nb (2750 K), solidify preferentially in the primary dendritic areas and lower melting point elements Ti (1941 K), Zr (2128 K), and Hf (2506 K) formed in the inter-dendritic areas [26]. The flower-like structures can be attributed to solid-state phase transformations or the development of fine-scale compositional modulations in the inter-dendritic regions. The microstructural evolution in these HEAs is highly sensitive to the cooling rate, and high cooling rates promote fine dendritic structure and may influence the evolution of these intricate morphologies.

### 3.2. Electrochemical Impedance Spectroscopy

**Figure 2:**
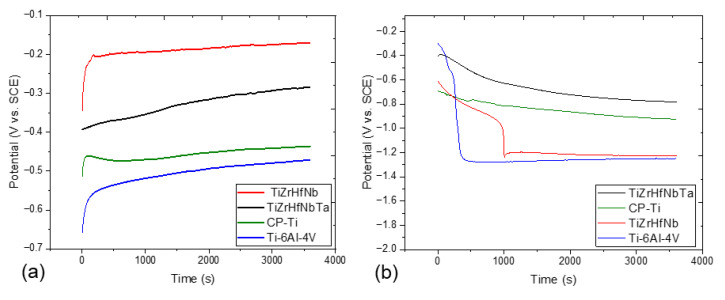
OCP plots of all samples in (**a**) AS and (**b**) ASF solution.

The OCP measurements in Figure 2 confirm that the optimal OCP value is −0.2 V (vs. reference) in TiZrHfNb, representing the best corrosion protection within the AS solution as well as within the ASF solutions. An initial rapid climb in OCP for TiZrHfNb indicates a spontaneous and very fast formation of a very protective passive oxide film [27]. This passive film offers effective protection from the aggressive chloride ions in AS and more corrosive fluoride ions in ASF. TiZrHfNbTa maintains stable passivity over a period, confirming it to be the strongest material among the samples tested under both conditions. It is distinct from TiZrHfNb, which shows a relatively resistant behavior with OCP of about −0.4 V in AS, which shifts in the positive direction with advancing passivation [28]. However, the reduced passivation rate compared to TiZrHfNbTa could be indicative of a passive film with reduced thickness or lower stability due to compositional effects. In ASF, TiZrHfNb shows a sharp drop in OCP, which indicates poor resistance and failure to maintain passivity against fluoride attack.

The TiZrHfNbTa sample shows successive development of OCP from −0.45 V to −0.35 V in AS, typical of the development of a steady state Ti oxide passive layer well recognized for corrosion resistance at physiological conditions [29]. In the ASF solution, CP-Ti shows better corrosion resistance than TiZrHfNb, but with an unstable potential. The elevated behavior is also due to the natural stability and fluoride resistance of the Ti oxide film, as it works superior to the comparatively less protective TiZrHfNb film under fluoride treatment. Ti-6Al-4V has the lowest OCP in both solutions at around −0.65 V, suggesting the highest susceptibility to fluoride- and chloride-induced corrosion [30]. Despite initial passivation, Ti-6Al-4V final position at most-negative potential guarantees poor passive film stability, perhaps undermined by aluminum and vanadium constituents that detrimentally affect oxide adhesion under corroding conditions [31]. TiZrHfNbTa is the most electrochemically stable and resistant to corrosion in both AS and ASF solutions and is therefore an optimal choice for long-term performance under biomedical or structural conditions in environments that are chloride- and fluoride-containing [32].

**Figure 3:**
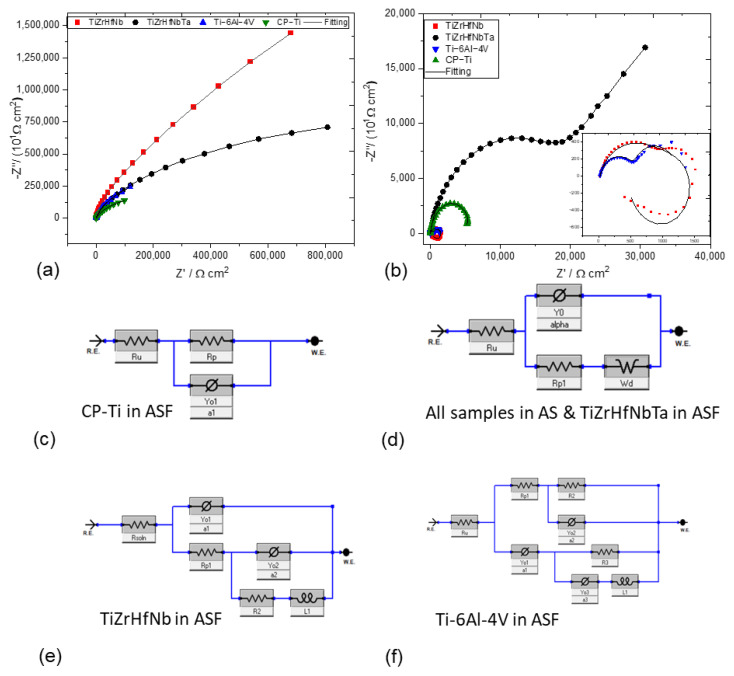
Nyquist plots of all samples in (**a**) AS and (**b**) ASF solution, along with proposed equivalent circuit models for EIS data fitting for: (**c**) CP-Ti in ASF, (**d**) all samples in AS and TiZrHfNbTa in ASF, (**e**) TiZrHfNb in ASF, and (**f**) Ti-6Al-4V in ASF solution.

EIS spectra shown in Figure 3 compare the electrochemical behavior of TiZrHfNbTa, TiZrHfNb, CP-Ti, and Ti-6Al-4V alloys when subjected to testing solution AS and ASF. Nyquist plots exhibit incomplete and depressed semicircles, suggesting that the electrochemical behavior at these electrode–electrolyte interfaces is predominantly controlled by charge transfer processes, although a slight contribution from diffusion processes remains evident in certain cases. Several equivalent circuit models (ECM) were employed to appropriately fit each set of experimental data, and the resulting fitting parameters are tabulated in Table 3 and Figure 3c–f [33]. The electrochemical parameters of EIS fitting provide additional information on the corrosion behavior of the materials. Solution resistance (*R_u_*), the same for all samples, indicates no changes in electrolyte conductivity [34]. *R_p_*, or polarization resistance, is the most significant parameter of corrosion protection—the greater the *R_p_*, the more effective the corrosion protection [35]. *Y*_0_ and *a* also describe the capacitive behavior of the interface, and deviations from ideal capacitor behavior suggest surface inhomogeneity or defects in the passive film, respectively [36]. Warburg impedance (*W_d_*) is employed to describe the “knee” frequency at which the Nyquist plot shows a slope change, generally indicating the occurrence of a diffusion process. Such a knee is typically found at low frequencies and is related to diffusion-controlled processes like ion transport via the electrolyte or solid phase [37].

In the AS solution shown in Figure 3a, all the samples tested were fitted with the optimal-fit standard Randles circuit model complemented by a diffusion component (Warburg impedance) [38]. The Warburg appearance suggests that the corrosion process in all samples was under partial control of ion diffusion through the passive layer, which is indicative of a protective oxide film with some mass transport restrictions [39]. This is typical for the overall stability of the passive films in the chloride-containing AS solution, where charge transfer and diffusion-controlled phenomena [40] dominate corrosion resistance. On the other hand, the ASF solution, as in Figure 3b, significantly altered the electrochemical behavior of the samples, as indicated by the need for different circuit models to describe each material, TiZrHfNbTa. This shows TiZrHfNbTa’s high resistance to fluoride attack as the Ta oxide layer did not crack but continued to remain protective throughout. For CP-Ti, even a simple Randles model with no additional components was able to model the EIS curve, which means that despite the breakdown of the passive film, it did not exhibit extensive diffusion limitations or local corrosion behavior when being exposed to fluoride media. In contrast, TiZrHfNb required the addition of an inductive element (*L*) to fit the experimental data adequately. The appearance of an inductive response is generally associated with the adsorption of some oxide layer or any corrosion layer on the surface of the alloy [41]. This indicates that TiZrHfNb was highly susceptible to fluoride-induced degradation, with the passive layer being unstable and the sites of localized corrosion most likely to be initiated. Similarly, Ti-6Al-4V possessed the most complex ECM, which required not only an inductive element but also an additional loop for two time constants. This multi-step model shows extensive passive layer degradation and formation of multiple corrosion sites that lead to strongly localized attack and increased degradation in the presence of fluoride ions. Inductive response and second time constant show the simultaneous occurrence of surface film degradation and re-passivation attempt, which indicates poor resistance of the material in these aggressive conditions.

In the AS solution, EIS parameters calculated from Figure 3a are in conformity with OCP behavior, very close values of *R_u_* ranging from 24.35 to 25.69 Ω were derived for all the samples. The maximum value of *R*_*p*1_, equal to 3634.3 Ω, was obtained for TiZrHfNb, followed by TiZrHfNbTa with 3269.5 Ω. CP-Ti possessed a moderate value of *R*_*p*1_ equal to 2822.5 Ω, while the minimum *R_p_*_1_ equal to 2500.7 Ω was obtained for Ti-6Al-4V, reconfirming its inferior corrosion resistance.

Although TiZrHfNb had the largest *R*_*p*1_ calculated from Figure 3a, and more positive OCP and its better *a*_1_ values support the conclusion that TiZrHfNb has better long-term stability. CP-Ti had *Y*_0_ of 3.54 × 10^−6^ S·s^a^, which represents good but not outstanding stability, and Ti-6Al-4V passive film remained the most imperfect with smaller *a*_1_ of 0.81 and greater *W_d_*, which bears witness to the presence of diffusion-controlled degradation. Electrochemical behavior of TiZrHfNb and TiZrHfNbTa alloys in the AS solution can easily be discriminated based on the calculation of their Warburg diffusion coefficients (*W_d_*), representing diffusion-controlled processes in the passive films. *W_d_* for TiZrHfNb was 1.23 × 10^−6^ S·s^½^, whereas TiZrHfNbTa was about an order of magnitude higher at 12.3 × 10^−6^ S·s^½^, revealing that TiZrHfNbTa experiences ten times more diffusion-related activity upon electrochemical testing. A narrower *W_d_*, such as in the case of TiZrHfNb, would rather be found with a denser and more compact passive oxide film with greater inhibitive ability to ionic transport and therefore, better corrosion resistance. However, the larger *W_d_* value in the case of TiZrHfNbTa suggests its passive film to be less defective or less porous in nature and supports quick ionic migration and therefore smoother degradation. This may be because the inclusion of Ta, as a stable oxide (Ta_2_O_5_) was created, would be likely to break up the homogeneity of the oxide film developed with Ti, Zr, Hf, and Nb. These disturbances cause inhomogeneous oxide growth, phase segregation, or electrochemical mismatches that embrittle the film in a highly corrosive, fluoride-free environment like AS.

When exposed to the ASF, the electrochemical performance of the materials changed considerably, as indicated in the second EIS plot Figure 3b. The fluoride ions have a pronounced influence on the stability of the passive oxide films in a deteriorating manner. *R_u_* values ranged from 34.37 Ω to 36 Ω, and TiZrHfNbTa showed the highest *R_u_* of 35.75 Ω, with higher resistance to ionic conduction, whereas CP-Ti and TiZrHfNb showed moderate values. TiZrHfNbTa exhibited the best corrosion resistance with the maximum *R*_*p*1_ value of 3569.9 Ω, which is in favor of its characteristic of being able to sustain an intact and stable passive film even under corrosive fluoride media. This kind of performance is favored by low *Y*_0_ (2.37 × 10^−6^ S·s^a^) and high *a* of 0.874, which reflects a relatively ideal capacitive response and dense, stable passive film. Somewhat unexpectedly, CP-Ti ranked second with an *R_p1_* of 2134.26 Ω, which shows good resistance and better performance than TiZrHfNb under these conditions. This is a result of the relatively stable Ti oxide passive layer with resistance to some fluoride. TiZrHfNb was severely susceptible to corrosion by fluoride, as indicated by the low *R*_*p*1_ of 1030.48 Ω. It indicates an unstable passive layer that was not resistant to fluoride attack. Also, it is extremely high *Y*_0_ (0.00103 S·s^a^), and other circuit elements (*L* = 530.34 H, *R*_2_ = 576.53 Ω, *Y*_02_ = 0.03 S·s^a^) indicate local corrosion, breakdown of passive film, and formation of unstable inner and outer oxide layers. Ti-6Al-4V performed the worst, with a very low *R*_*p*1_ of 661.46 Ω and the lowest *a*_1_ value of 0.84, again pointing to rapid degradation and passive film breakdown. The inductive loop (*L* = 427.2 H) in Ti-6Al-4V confirms the initiation of localized corrosion mechanisms, which are catastrophic for mechanical integrity. Briefly, the EIS test indicates that in the AS solution, TiZrHfNbTa possesses the highest corrosion resistance due to its stable passive film, followed by TiZrHfNb with relatively good resistance, and CP-Ti and Ti-6Al-4V possess good and poor resistance, respectively. The sequence totally reverses in fluoride-containing ASF solution due to the active nature of fluoride ions. TiZrHfNbTa exhibits the highest corrosion resistance, followed by CP-Ti, surprisingly outperforming TiZrHfNb due to the natural resistance of Ti to fluoride. TiZrHfNb performs badly due to breakdown of films and local attacks, and Ti-6Al-4V, as would be the situation with its vulnerability to being attacked by fluoride ions, has the poorest resistance. This comprehensive electrochemical research brings into perspective the highly superior appropriateness of TiZrHfNbTa for use in severe environments, particularly in uses such as chloride and fluoride ions, where other materials degrade very rapidly.

### 3.3. Cyclic Potentiodynamic Polarization

**Figure 4:**
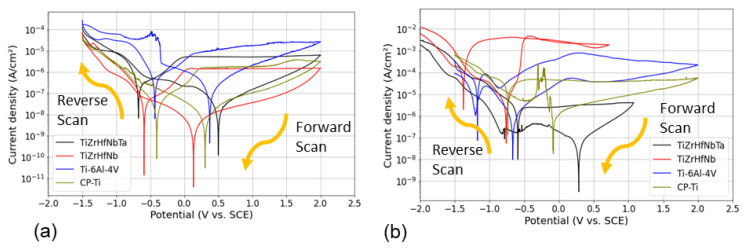
CPP plots of all samples in (**a**) AS and (**b**) ASF solution.

The CPP measurements in AS and ASF solutions were performed to evaluate the corrosion behavior of TiZrHfNb, TiZrHfNbTa, CP-Ti, and Ti-6Al-4V alloys, as shown in Figure 4. The most significant electrochemical parameters investigated are the corrosion potential (*E_corr_*), corrosion current density (*i_corr_*), passivation potential (*E_pass_*), pitting potential (*E_pit_*), and the active-to-passive transition potential (*E_a/c_*) are derived from the plots and presented in Table 4. They are utilized for pitting and passive properties determination of the alloys investigated.

The initial rapid anodic dissolution took place as metal elements oxidized to produce metal ions (M^n+^) and diffused into solution, causing a steep current density increase due to the absence of a protective oxide layer. As the potential rose, passivation occurred, and a stable protective layer was formed that reduced current density. Negative hysteresis following the reverse scan showed quick repassivation and enhanced corrosion resistance [42]. TiZrHfNb showed the optimal corrosion resistance in the AS solution among the tested samples, as can be seen from Figure 4a. As a multi-principal element alloy, it was electrochemically stable with the lowest *i_corr_* of 26.98 nA·cm^−2^ and comparatively noble *E_corr_* of 147.70 mV. The alloy exhibited a passivation potential of 0.2 V and a very high pitting potential of 2.0 V, indicative of premature passivation and excellent localized corrosion resistance. The enhanced stability is attributed to the formation of a stable and protective HfO_2_ passive film, which can effectively suppress anodic dissolution [43]. Although TiZrHfNbTa also exhibited acceptable corrosion resistance with *i_corr_* of 18.38 nA·cm^−2^ and *E_corr_* of 300.10 mV, its low *E_pass_* of 0.1 V and lower *E_pit_* of 1.8 V suggested a less stable passive film than TiZrHfNb. While Ta addition promoted passivation through the formation of stable Ta_2_O_5_, the EIS tests confirmed that the Ta_2_O_5_ layer experienced greater diffusion limitations in AS solution, potentially inhibiting ion transport and affecting the dynamic stability of the passive film [44]. In contrast, the formed passive layer on TiZrHfNb exhibited better electrochemical kinetics, maintaining more stable passivation behavior. The large *E_pit_* and *E_pass_*, as well as EIS-derived diffusional behavior, indicate that TiZrHfNb is more resistant to general and localized corrosion in AS solution. In contrast, CP-Ti had a significantly higher *i_corr_* of 84.20 nA·cm^−2^ and *E_corr_* of 275.20 mV and was more susceptible to corrosion despite its capacity to passivate. Its lower *E_pass_* (0.05 V) and *E_pit_* (1.2 V) indicated susceptibility to localized corrosion, i.e., that its passive film is not as stable in AS solution. Ti-6Al-4V with *i_corr_* of 71.91 nA·cm^−2^ and *E_corr_* of 289 mV showed better passivation behavior than CP-Ti. Its relatively high *E_pass_* of 0.3 V showed its ability to form a protective oxide film, but its low *E_pit_* (1.6 V) showed poor resistance to localized corrosion.

A comparative study of standard electrode potentials (vs. SHE) of the elements constituting it for determining their relative nobility is +0.1 V for Ta, −0.8 V for Nb, −1.45 V for Zr, −1.63 V for Ti, and −1.55 V for Hf. From this, Ta and Nb are relatively more noble and must contribute to stabilizing the passive film. Ti, Hf, and Zr, although less noble, yield steady and thick oxide films (TiO_2_, HfO_2_, and ZrO_2_) that encourage overall corrosion resistance. The *E_corr_* values obtained in our study are similar to those reported in the literature for Ti–Nb–Zr and Ti–Ta alloys under similar conditions, but the *i_corr_* values tend to be lower, indicating a higher passivity in our multicomponent system.

In the aggressive ASF solution, all the alloys exhibited greater corrosion rates due to the very active anodic environment. The behavior has been shown in Figure 4b. However, among them, TiZrHfNbTa was more corrosion-resistant and stable, and the most electrochemically resistant material under these conditions. This superior performance is attributable to the incorporation of Ta in its alloy, enhancing passivation and localized corrosion resistance [45]. TiZrHfNbTa possessed the lowest *i_corr_* (40.70 nA·cm^−2^), indicating lower material dissolution, and the highest *E_pit_* (1.4 V), which justifies its superior resistance to localized attack. Although its *E_pass_* of 0.3 V was modest, it was sufficient to trigger stable passive film formation, thereby preventing excessive surface degradation. In addition, the electrochemical stability of TiZrHfNbTa in the ASF solution was supported by its relatively more noble *E_corr_* of 285.20 mV. The formation of high dielectric strength and low-solubility Ta-rich oxide film (TaO_5_) is primarily responsible for improved performance of TiZrHfNbTa [46]. Unlike Ti-based alloys, which are more prone to local attack, the protective film not only improves passivation but also resists breakdown under aggressive conditions enriched with fluoride and chlorides [47]. TiZrHfNb exhibited extremely high negative *E_corr_* (−650.0 mV) compared to TiZrHfNbTa, with a greater tendency for uniform corrosion. Nevertheless, TiZrHfNb exhibited the highest *E_pit_* (1.1 V) and thus the highest localized corrosion resistance. CP-Ti, although it exhibited good passivation behavior, had enormous localized corrosion owing to the exceedingly high value of *i_corr_* (74.36 nA·cm^−2^) and was thus less reliable with longer exposure. Ti-6Al-4V exhibited unstable passivation with a lower *E_pit_* of 0.5 V and hence was even less stable under ASF conditions.

TiZrHfNbTa possessed enhanced corrosion resistance in AS and ASF solutions due to its lower *i_corr_*, improved *E_pit_*, and greater passive film stability. Ta addition electrochemically stabilized and significantly improved the passive layer, rendering TiZrHfNbTa an extremely suitable high-performance application material with superior corrosion resistance. Ta and Nb additions and synergy caused stabilization of the passive film and enhanced the resistance against localized attack [48]. Passivation of TiZrHfNb was good but less efficient compared to the case of TiZrHfNbTa. CP-Ti and Ti-6Al-4V were more prone to local corrosion due to unstable passive films, limiting their usage in aggressive corrosive environments. TiZrHfNbTa possessed the highest potential as a corrosion-resistant alloy and performed better than other alloys in the formation of a strong and stable passive layer.

### 3.4. Mott–Schottky

The Mott–Schottky analysis provides valuable information on the electronic properties of passive films formed on Ti alloys and HEAs under different environmental conditions [49]. Donor density (*N_D_*) and semiconductor type (p-type or n-type) calculated by fitting the data provide important information on the influence of environmental parameters, e.g., fluoride ions and physiological solutions, on the stability and corrosion resistance of these passive films. The analysis is applied to gain a profound understanding of the semiconductor properties of passive films formed on metal surfaces depending on the inverse square of space charge capacitance (*C*) from the applied potential (*E*) [50]. The basic equations that govern the M-S analysis are as follows [51]:

*N_D_* = (2 × *ε* × *ε*_0_)/(*e* × *Slope*)

where *ε*_0_ = 8.854 × 10^−14^ F/cm (vacuum permittivity), *ε* = dielectric constant of the material, *e* = 1.602 × 10^−19^ C (elementary charge), and slope values are provided for each alloy. The *ε* used for HEA is 1000, Ti is 18.1, and Ti-6Al-4V is 18.1. *E* is the flat band potential (V), *k* is Boltzmann’s constant (1.38 × 10 J/K), and *T* is the absolute temperature (K).

**Figure 5:**
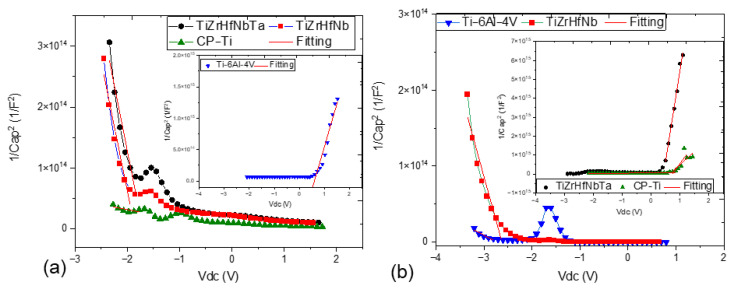
Mott–Schottky plots of all samples for (**a**) AS and (**b**) ASF solutions.

The general semiconductor character of the four samples has been demonstrated through the Mott–Schottky plots in AS and ASF solution in Figure 5a and b, respectively. Negative slopes for both TiZrHfNbTa and TiZrHfNb, as depicted in Figure 5a, are evidence that they are p-type semiconductors. Their p-type behavior is because the cation vacancy is dominant as an acceptor site in passive films. The high vacancy concentration facilitates the easy formation of a dense and protective passivating layer that effectively suppresses penetration by aggressive ions such as chlorides [52]. As clearly seen, TiZrHfNb possesses the best passive film performance by virtue of moderate defect density with a donor density of −2.6 × 10^20^ cm^−3^, which ensures film stability as well as severe corrosion resistance. TiZrHfNbTa also exhibits p-type behavior with a lower concentration of donors of −1.8 × 10^19^ cm^−3^, which indicates good passivation but is comparatively less stable than TiZrHfNb due to surface/interface electrolyte in alloy composition influence over vacancy formation and oxide compaction. On the other hand, CP-Ti and Ti-6Al-4V exhibit M-S curves of the positive slope characteristic of n-type semiconductor behavior dominated by oxygen vacancies and interstitial cations as electron donors. The donor concentration for CP-Ti is 8.1 × 10^19^ cm^−3^, demonstrating highly developed electron carrier activity in the oxide film. These types of defects diminish the passive layer, and Ti can experience localized corrosion in environments that contain chloride [53]. Ti-6Al-4V has an even bigger *N_D_* of −4.7 × 10^20^ cm^−3^ with a very defective passive film that tends to deteriorate. The very high oxygen vacancy density of Ti-6Al-4V facilitates ionic conductivity and ingress of chloride ions, and hence greater susceptibility to pitting and localized corrosion [54].

The semiconductor nature of the passive films is altered when exposed to the more corrosive fluoride-containing ASF solution. TiZrHfNbTa is converted to n-type conductivity, as can be seen from Table 5, suggesting a dominance of oxygen vacancies over cation vacancies. On the other hand, TiZrHfNb still maintains its p-type characteristic, suggesting that cation vacancies remain a significant contribution to the latter’s passive film growth [55]. The steeper slope in Mott-–Schottky plots in Figure 5b for the two alloys under ASF conditions suggests increases in defect densities with fluoride-induced interruptions in the oxide films. Despite that, TiZrHfNbTa, with an *N_D_* of 0.11 × 10^20^ cm^−3^ in ASF, still maintains the most stable passive film. Its relatively low defect density has sustained the highest corrosion resistance even under fluoride attack. The technical advantage of TiZrHfNbTa is that it can form a dense, protective passive film with resistance to fluoride ion penetration owing to its HEA matrix and uniform elemental distribution, improving oxide film integrity and stability [56].

Conversely, TiZrHfNb has an *N_D_* of -2.3 × 10^20^ cm^−3^ in ASF, suggesting a trend towards higher defect densities. This further means that the fluoride ions destabilize the passive film more in TiZrHfNb compared to TiZrHfNbTa. The passive layer of TiZrHfNb is not as stable despite still offering good protection due to fluoride-assisted cation vacancy creation, enhancing ionic transport paths, and lowering corrosion resistance [57]. However, Ti shows improved stability in ASF compared to AS with a lower *N_D_* of 0.05 × 10^20^ cm^−3^. This decrease in oxygen vacancies explains why the passive film of Ti is denser or less conductive with better corrosion performance when exposed to fluoride than TiZrHfNb under that condition. The reason lies in the fact that Ti possesses the renowned characteristic of creating stable and fluoride-resistant oxide films that reduce electron donor sites and enhance passivity. However, Ti-6Al-4V remains the weakest sample, with *N_D_* worsening to −4.8 × 10^20^ cm^−3^ in ASF. The addition of Al and V in Ti-6Al-4V significantly undermines the quality of the passive film, causing greater defects and promoting localized corrosion at an accelerated rate. The transition towards mixed n-type and p-type behavior for Ti-6Al-4V indicates severe degradation and breakdown of the passive film under fluoride attack, confirming its high susceptibility to aggressive media [58].

TiZrHfNbTa remains consistently good in corrosion resistance, and this owes much to its exceptionally balanced defect density and strong passive film stability. Its HEA structure makes it easier to achieve a homogenized microstructure and oxide growth, enhancing stability against chloride and fluoride attacks. This enhanced performance is of technical significance since TiZrHfNbTa exploits lattice distortion effects, sluggish diffusion, and thermodynamic stability in HEAs, all of which contribute to a more protective and long-lasting passive film [59]. TiZrHfNb, while having good performance in AS, is less resistant in ASF due to amplified fluoride-induced defects. CP-Ti exhibits improvement in ASF, being superior to TiZrHfNb, owing to the resistance of its native TiO_2_ passive film to fluoride attack. Ti-6Al-4V, with the highest constant defect density, is the least resistant in both solutions, being unable to form a stable passive film. The *N_D_* calculations confirm lower values to be correlated with higher passive film stability and lower corrosion susceptibility, as exhibited by the performance of TiZrHfNbTa. On the other hand, the high *N_D_* of Ti-6Al-4V always signifies poor film quality and vulnerability.

### 3.5. Potentiostatic Polarization

The PSP plots of TiZrHfNb and TiZrHfNbTa within AS and ASF solutions provide useful information concerning their passivation kinetics, electrochemical stability, and corrosion protection against localized corrosion in corrosive environments [60]. Wide differences in passive film forming and degrading mechanisms of these HEAs under anodic polarization conditions exist in the resulting current density-time profiles obtained using potentiostatic tests. The subsequent AFM analysis also corroborates these electrochemical results by morphology alteration on the corroded surfaces.

**Figure 6:**
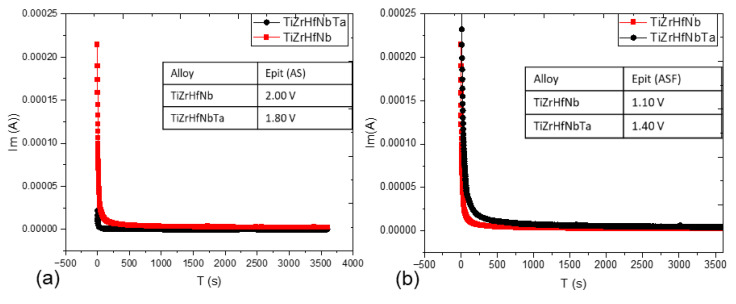
PSP plots of HEA samples for (**a**) AS and (**b**) ASF solution.

TiZrHfNb and TiZrHfNbTa both exhibited, in the AS solution Figure 6a, an initially high anodic current density, which fell rapidly with time, a typical indicator of passive film formation. TiZrHfNb showed a significantly lower steady-state current density compared to TiZrHfNbTa, which reflects a more protective and stable passive film. The relatively stable current response of HEAs with minimal fluctuation suggests a dense and adherent oxide layer with reduced defect density, where localized corrosion initiation is less probable [61]. Conversely, TiZrHfNbTa exhibited a larger steady-state current density with minute fluctuations, which indicates a lower density passive layer that may feature localized defects or microstructural inhomogeneities and therefore, anodic dissolution at higher rates [62]. The same is indicated through AFM observation following PSP, too. With AS exposure, TiZrHfNbTa recorded a decrease in surface roughness from 6.49 nm to 2.58 nm, indicating that the corrosion process led to smoothing of the surface and not in the loss of significant material. This indicates that the passive film thickened and stabilized with increasing time, preventing further corrosion propagation. On the other hand, TiZrHfNb exhibited hardly any increase in roughness from 607.44 nm to 1.53 nm, confirming that its passive layer remained in good shape with hardly any degradation. The AFM scans are smooth and defect-free, verifying the high corrosion resistance of TiZrHfNb in AS.

The ASF solution, which is chemically aggressive owing to the presence of complex ionic species, showed considerably higher initial current densities for both alloys than AS depicted in Figure 6b. This reflects higher electrochemical activity and lower initial stability of passive films. TiZrHfNb showed oscillations of high amplitude of current density, reflecting instability of the passive layer and continued dissolution-repassivation cycling. Oscillatory current response suggests a localized mechanism of breakdown, such as pitting or crevice corrosion due to chloride or other potent anions in ASF [63]. TiZrHfNbTa exhibited more even and gradual reduction in current density, stabilizing at a much lower value than TiZrHfNb. This trend shows that TiZrHfNbTa forms a more stable and passive film, which can be enriched with stable oxides such as HfO_2_, TiO_2_, Nb_2_O_5_, and Ta_2_O_5_, which are effective in suppressing aggressive ion penetration and enhancing local corrosion resistance [64]. AFM observations after PSP testing also validate these trends, where significant differences in surface topography between the two alloys are observed. TiZrHfNb exhibited a high increase in roughness to 106.77 nm with extensive pitting and deep valleys, supporting extensive localized attack and accelerated material loss in ASF. The severe breakdown of passivation indicates that TiZrHfNb’s passivating film was destabilized by fluoride ions, leading to damaging pit propagation. On the other hand, TiZrHfNbTa exhibited a more controlled increase in roughness to 50.17 nm, indicating a more stable process of passivation. The roughening was a sign of the formation of a secondary protective film, which could be fluoride-stabilized, that suppressed excessive material loss and facilitated long-term corrosion resistance [65]. Compared with TiZrHfNb, TiZrHfNbTa’s ASF passive film was more stable, resisted aggressive ion-induced attack, and maintained a protective barrier over the course of time. TiZrHfNbTa’s electrochemical activity within ASF was appreciably augmented due to its greater stability of passivation and local attack resistance. The reduced current density oscillations indicate a lower dissolution rate and better overall corrosion resistance under fluoride-rich conditions [66]. TiZrHfNb, in contrast, had severe pitting and passivation layer instability, making it less suitable for such conditions. While TiZrHfNb was stable in AS, its activity in ASF was limited due to widespread local corrosion. These findings confirm TiZrHfNbTa as the best candidate for high-corrosion conditions in chemically complex media, particularly fluoride media. Morphological and electrochemical investigations highlight the significance of intact passive film for determining long-term corrosion behavior, revealing that TiZrHfNbTa’s excellent passive film stability and resistance to localized corrosion render it an ideal candidate for dental implant applications in hostile physiological media.

### 3.6. Post Corrosion AFM Observation

**Figure 7:**
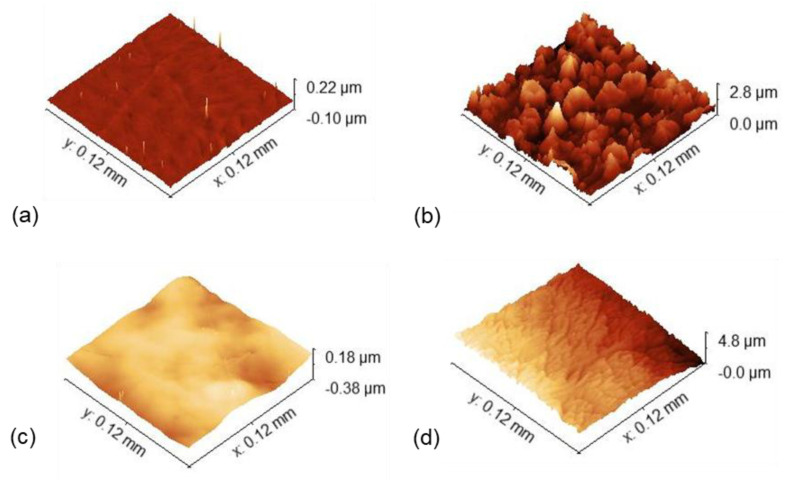
AFM images of HEA samples after PSP tests: (**a**) TiZrHfNb and (**c**) TiZrHfNbTa in AS solution, (**b**) TiZrHfNb and (**d**) TiZrHfNbTa in ASF solution.

AFM image of TiZrHfNbTa in Figure 7 illustrates a surprising decrease in surface roughness, as the Ra value decreases from 6.49 nm to 2.58 nm, after being exposed to the PSP test in AS solution (Table 6). This shows that corrosion has not invaded the material aggressively but instead led to surface smoothing, which is a consequence of the protective oxide layer. The AFM scan is a smoother surface with reduced peak-to-valley variations, indicating that the corrosive environment has favored passivation over uncontrolled material loss. Electrochemically, this is the preferred result, since the smooth and intact passive layer can act as a barrier to further corrosion. This agrees with previous studies on the electrochemical behavior of transition metal oxides in acidic media, where passivation accounts for the reduction in corrosion rates [67]. Passive film thickening of TiZrHfNbTa in AS shows that the material stabilizes in very slightly aggressive acidified media. AFM micrograph of TiZrHfNb exhibits barely any roughening of the surface as Ra rises from 0.61 nm to 1.53 nm, when exposed to AS solution. Although the barely noticeable roughening takes place, the surface is extremely smooth, and it is not clear that corrosion has damaged the material in a measurable way. AFM images show only slight morphological change without pitting or structural degradation to a very large degree. This signifies that TiZrHfNb exhibits better corrosion resistance in AS, with an intact passive film without extensive degradation. Electrochemically, this is consistent with the results of impedance spectroscopy that show lower corrosion currents and polarization resistance for materials with stable passive films. The slight increase in roughness testifies that the oxide layer over the material is intact, in agreement with the improved performance of TiZrHfNb in an acidic environment.

AFM image in Figure 7c of TiZrHfNb after corrosion in ASF solution demonstrates a phenomenal rise in surface roughness from 0.61 nm to 106.77 nm, indicating colossal surface damage. The topography shown in the scan reveals deformed and turbulent topography with extremely deep pits and sharp peaks, suggesting highly localized corrosion or pitting. The higher reactive Ti, Zr, Hf, and Nb oxides were not able to withstand fluoride attack, leading to breakdown of the passive film and exposure of the alloy matrix to the corrosive environment. This led to micro-electrochemical cells that formed progressively deepening pits, leading to extensive material loss and mechanical compromise. Unlike TiZrHfNbTa, no protective re-passivation is observed, and the surface remains dominated by extensive localized damage. Formation of soluble fluoride complexes sped up pitting and roughening, demonstrating worse corrosion resistance of TiZrHfNb in ASF. AFM scan of TiZrHfNbTa following corrosion in ASF solution shows modest roughness augmentation, as Ra increases from 6.49 nm to 50.17 nm. It is a low and controlled level of increase in roughness, and it confirms that TiZrHfNbTa resisted heavy surface deterioration with only mild and uniform surface transformation instead of extensive localized corrosion. The AFM picture indicates a flat topography with no typical peaks or valleys, confirming the existence of a protective passive film enriched with stable Ta_2_O_5_ that hinders the penetration of fluoride, as shown in Figure 7d. Under active fluoride ions (F^−^) conditions, the solid Ta_2_O_5_ layer exhibited extreme resistance towards complexation and dissolution and curbed any future localized attack [68]. Absence of deep pits or serrations testifies that TiZrHfNbTa was able to maintain its structural integrity under the ASF conditions, which testifies that general surface passivation was better than pitting, which suggests a dominance of general surface passivation. This action also emphasizes the protective function of Ta in improving corrosion resistance by stabilizing the passive film against fluoride-induced breakdown [69].

### 3.7. Post Corrosion SEM Observation

**Figure 8:**
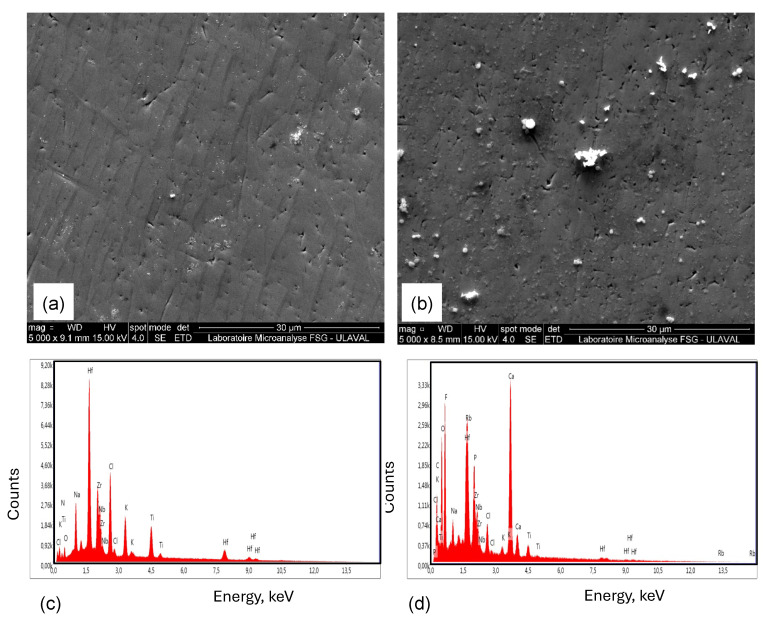
SEM micrographs of HEA samples after PSP tests: (**a**) TiZrHfNb and (**b**) TiZrHfNbTa, and EDX spectra of (**c**) TiZrHfNb and (**d**) TiZrHfNbTa in AS solution.

Figure 8a presents the surface morphology of TiZrHfNb alloy following PSP exposure in AS and demonstrates a quite flat and comparatively well-preserved microstructure. Fine-grained, compact alloy structure does not exhibit significant signs of degradation but only minor etching with some scattered dark spots, most probably indicating early oxide formation or localized surface reaction. There was no evidence of deep pitting, micro-cracking, or severe surface attack, revealing the protective nature of a stable and moderately passivating film. Regardless of the existence of chloride ions arising from both NaCl and CaCl_2_·2H_2_O present in the AS solution, this oxide film is determined to be strong enough [70]. That is because such action is mainly attributed to the mixed oxides synergy of Zr, Nb, and Hf, which results in a general type of corrosion. The homogeneous EDX analysis shown in Figure 8c and Table 7 further proves the direction towards the existence of a chemically stable continuous film as well [71]. The principal components (Ti, Zr, Hf, and Nb) are still uniformly distributed with no trace of elemental loss or localized leaching. These results again confirm that TiZrHfNb possesses very good surface integrity and corrosion resistance in AS, with very good resistance to chloride-induced passive layer breakdown [72].

Figure 8b reveals the surface morphology of TiZrHfNbTa alloy after exposure to AS, and it exhibits increased heterogeneity and small signs of localized corrosion. The mottled appearance of the image is enhanced by the fact that there are many dark etched regions and surface irregularities, which seem to point to breakdown or instability in the passive film. In contrast, the process of the introduction of Ta is envisioned to enhance corrosion resistance due to the production of stable Ta_2_O_5_; hence, electrochemical inhomogeneities point towards galvanic interactions or non-uniform Ta distribution that might provide possible pathways for localized corrosion [73]. The respective EDX spectrum shown in Figure 8d and Table 8 substantiates this with increased intensities of Ca, Cl, and Na—typical AS corrosion products, coupled with inhomogeneous oxygen distribution. Ta is also present, but its protective effect in this case is low, as confirmed by EIS testing. The occurrence of secondary species like Rb and P indicates contamination or complex surface reactions, again confirming the observation of increased surface reactivity and corrosion evolution in TiZrHfNbTa under AS conditions.

**Figure 9:**
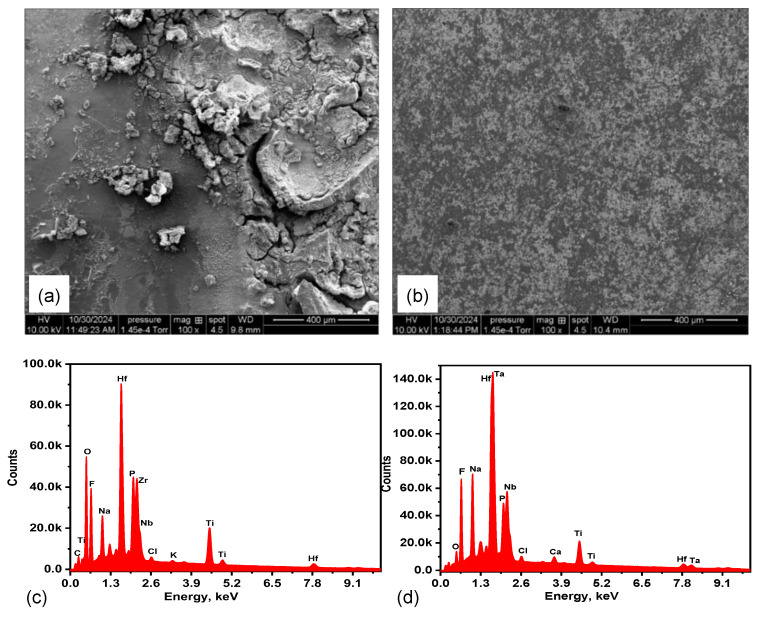
SEM micrographs of HEA samples after PSP tests: (**a**) TiZrHfNb and (**b**) TiZrHfNbTa, and EDX spectra of (**c**) TiZrHfNb and (**d**) TiZrHfNbTa in the ASF solution.

Figure 9a shows the composite assessment of TiZrHfNb under ASF with the addition of fluoride ions. The SEM micrograph is indicative of extensive deterioration of the material, especially towards the left-hand side, with flake or delaminated structures. Widespread intergranular attack and pitting indicate the rupture of TiZrHfNb passive film when exposed to fluoride [74]. Some surfaces are smoother, still contain surviving passive film, but huge areas underwent extensive deterioration. These results are supported by the EDX spectrum in Figure 9c and Table 8, with high oxygen (16.73 wt%) being the cause of corrosion product and degradation of a passive layer. Aggressive fluoride ion participation is established by high fluoride (9.22 wt%). Although Ti (13.49 wt%) and Zr (25.70 wt%) are present, their protective oxide fails to prevent fluoride attack. Trace amounts of chlorine indicate possible combined effects of chlorides and fluorides, causing additional material loss and deterioration.

Elemental mapping detects the presence of fluoride in severely degraded regions, as anticipated by regions of fluoride ion attack. The oxygen increase in the regions confirms passive layer failure and subsequent oxidation. Propagation of corrosion suggests microstructural influence, where certain phases are easily susceptible to failure. The excessive pitting and material loss substantiate that despite the presence of Ti and Zr, TiZrHfNb cannot maintain stable passivation against fluoride. Comparing the two HEAs, there is more Ti (13.49 wt%) and Zr (25.70 wt%) in TiZrHfNb, but less effective in ASF with fluoride ions. TiZrHfNbTa, with 30.61 wt% Ta content, is superior in corrosion resistance. Ta possesses the unique characteristic of developing a stable and resistant oxide layer (Ta_2_O_5_), and therefore, it is less susceptible to fluoride ion breakdown. Ta oxides are different from Ti or Zr oxides, which are more soluble in fluoride. In addition, TiZrHfNb’s high oxygen content reflects thick but ineffective oxides that fail against fluoride attack, leading to severe corrosion, as observed in previous work on TiZrHfNb in another fluoride-containing electrolyte [75].

The SEM micrographs of TiZrHfNbTa, as shown in Figure 9b, exposed to simulated ASF containing fluoride ions, exhibit generally enhanced surface stability with fewer aggressive corrosion features. A large bright pit in the center of the micrograph, and a little roughness around it, but no wide-ranging material dissolution exists. The small pits and microcracks can be observed, but less extreme, indicating the passive layer of TiZrHfNbTa, Ta-rich, tends to be protective overall. The corrosion appears to be in check, as enormous components remain smooth, suggesting that fluoride ions possess limited attack ability against the more robust passive layer. Ta is known for its excellent corrosion resistance in aggressive fluoride environments due to the formation of stable Ta pentoxide (Ta_2_O_5_), which is resistant to dissolution and passivation. EDX elemental mapping of TiZrHfNbTa also reiterates decreased corrosive attack. Figure 9d and Table 8 show Ta-rich regions unchanged, while a slight local attack occurs elsewhere. The presence of Ta enhances the passivity of the passive layer so that severe deterioration is not allowed [76]. The selective leaching becomes less evident, and dark spots signify minimum loss of Ta, reiterating increased corrosion resistance due to the protective action of Ta oxides even in ASF with fluoride on long-term exposure.

Quantitative EDX analysis of TiZrHfNbTa, as shown in Figure 9d, also provides more insight into this behavior. Mean oxygen content (2.27 wt%) again reflects some passive layer but is not entirely representative because of potential measurement artifacts. High Ta content (30.61 wt%) contributes strongly to corrosion resistance because Ta forms fluoride-resistant oxides. Fluoride content (9.23 wt%) indicates exposure, but its contribution is less detrimental owing to Ta’s protective character. Hf (30.17 wt%) and Nb (15.71 wt%) contribute to passive film development, but Ta dominates fluoride aggression protection. Phosphorus (7.41 wt%) and sodium (7.33 wt%) are possible deposits from the simulated solution, while trace calcium (1.29 wt%) and chlorine (0.39 wt%) show traces of biomineralization and chloride activity. SEM imaging, EDX mapping, and elemental analysis collectively confirm that TiZrHfNbTa resists more effectively fluoride-induced pitting corrosion due to the enhanced resistance of Ta to fluoride. The ability of Ta to form a stable, adherent Ta_2_O_5_ hinders passive layer breakdown and controls localized attack. Although pitting cannot be avoided, it is contained, confirming TiZrHfNbTa’s superior performance against fluoride-mediated degradation compared to other HEAs.

Therefore, TiZrHfNbTa is superior in ASF, despite lower oxygen content, Ta offers long-term passive film integrity even in fluoride-containing environments. TiZrHfNb, despite the formation of more oxides, lacks this fluoride-specific resistance and hence suffers from aggressive pitting. The Ta resistance against fluoride ion attack makes TiZrHfNbTa a superior choice for such environments. In general, pitting and intergranular attack are dominant localized corrosion mechanisms triggered by fluoride ions. TiZrHfNb oxide layer degrades and creates less protective fluoride compounds, causing material loss, while that of TiZrHfNbTa is resistant to degradation through a passive layer with stability. The extended exposure to fluoride exposes TiZrHfNb to structural degradation, while TiZrHfNbTa is less degraded because of the higher chemical stability of Ta.

## 4. Discussion

**Figure 10:**
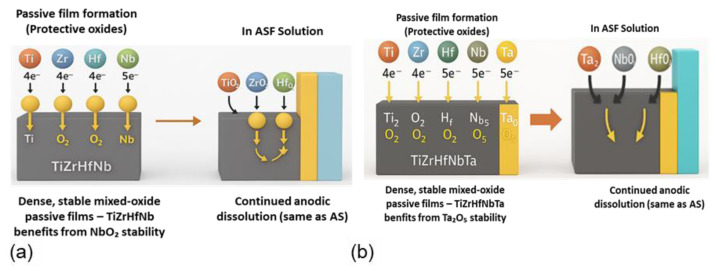
Suggested corrosion phenomenon for (**a**) TiZrHfNb and (**b**) TiZrHfNbTa in the ASF solution.

The corrosion resistance and electrochemical response of TiZrHfNb and TiZrHfNbTa in AS and ASF environments are dominated by passive film stability and ionic interaction at the solution–alloy interface as depicted in Figure 10. In the AS solution, alloying elements such as Ti, Zr, Hf, Nb, and Ta are anodically dissolved and subsequently passivated quickly by the formation of thermodynamically stable and insoluble oxide films (TiO_2_, ZrO_2_, HfO_2_, Nb_2_O_5_, and Ta_2_O_5_). The oxides are characterized by high dielectric constants and low solubility, leading to low current density and the formation of a compact, smooth passive film, as revealed in previous work on TiZrHfNb in a similar simulated body fluid electrolyte [77]. Furthermore, TiZrHfNbTa is greatly enhanced by the increased stability and denser nature of the Ta_2_O_5_ layer, with its protective function being higher than TiZrHfNb.

The overall passive film formation can be represented as follows:

M → M^n+^ + ne^−^ (1)

M^n+^ + H_2_O → MO_x_ + 2H^+^ (2)

When subjected to the ASF solution, with aggressive fluoride ions, there are complex electrochemical interactions. Fluoride ions attack the passive layers by forming soluble metal fluoride complexes such as [TiF_6_]^2−^, [ZrF_6_]^2−^, and [TaF_7_]^2−^. Despite the fact that complex formation is thermodynamically favorable with very negative Gibbs free energy, the Ta_2_O_5_ layer of TiZrHfNbTa exhibits significantly higher resistance to fluoride-induced degradation. This is due to its high lattice energy and kinetic stability, which retard the process of breakdown and minimize the extent relative to the Ti and Zr oxides in TiZrHfNb.

Microstructural inhomogeneities and oxide instability in TiZrHfNb lead to sudden soluble fluoride formation, surface roughening, pitting, and high material loss, as verified by SEM-EDX. In comparison with TiZrHfNbTa, with its relatively stable surface, slight pitting, and low metal ion leaching as the signature, it is more corrosion resistant in fluoride-rich environments. These mechanistic findings are consistent with electrochemical data (potentiostatic profiles, EIS) and surface analysis (AFM and SEM-EDX), demonstrating that the stability of the passive film, oxide solubility, and the metal–fluoride complexation tendency are significant factors in controlling corrosion behavior. Understanding these fundamental reactions is crucial to designing HEAs for biomedical and strongly corrosive conditions, and TiZrHfNbTa is the best choice among them due to its high passive film stability and fluoride resistance.

I. In the AS solution—mildly corrosive

Anodic metal dissolution (common to both alloys)

Ti → Ti^4+^ + 4e^−^ (3)

Hf → Hf^4+^ + 4e^−^ (4)

Nb → Nb^5+^ + 5e^−^ (5)

Ta → Ta^5+^ + 5e^−^ (only in TiZrHfNbTa) (6)

Passive film formation (protective oxides)

Ti^4+^ + 2H_2_O → TiO_2_ (s) + 4H^+^ (7)

Zr^4+^ + 2H_2_O → ZrO_2_ (s) + 4H^+^ (8)

Hf^4+^ + 2H_2_O → HfO_2_ (s) + 4H^+^ (9)

Nb^5+^ + 2.5H_2_O → Nb_2_O_5_ (s) + 5H^+^ (10)

Ta^5+^ + 2.5H_2_O → Ta_2_O_5_ (s) + 5H^+^ (11)

Dense, stable mixed-oxide passive films—TiZrHfNbTa benefits from Ta_2_O_5_ stability.

II. In the ASF solution—aggressive, fluoride-containing

Continued anodic dissolution (the same as in the AS);

Fluoride-induced passive film breakdown/complex formation;

Highly soluble, severe Ti loss in TiZrHfNb.

TiO_2_ (s) + 6F^−^ + 4H^+^ → [TiF_6_]^2−^ (aq) + 2H_2_O (12)

Moderate solubility contributes to pitting

ZrO_2_ (s) + 6F^−^ + 4H^+^ → [ZrF_6_]^2−^ (aq) + 2H_2_O (13)

Similar effect to Zr

HfO_2_ (s) + 6F^−^ + 4H^+^ → [HfF_6_]^2−^ (aq) + 2H_2_O (14)

Slower dissolution, offering mild protection

Nb_2_O_5_ + 10F^−^ + 10H^+^ → 2[NbF_5_] (aq) + 5H_2_O (15)

Kinetically sluggish, less favorable due to the high lattice energy of Ta_2_O_5_

Ta_2_O_5_ (s) + 14F^−^ + 10H^+^ ⇌ 2[TaF_7_]^2−^ (aq) + 5H_2_O (16)

## 5. Conclusions

In general, the TiZrHfNb and TiZrHfNbTa HEAs exhibited a far superior corrosion resistance compared to CP-Ti and Ti-6Al-4V alloys in both AS and ASF solutions. The experiment shows that the corrosion behavior of the TiZrHfNb and TiZrHfNbTa alloys significantly depends on the electrolyte environment. In the AS solution, the corrosion resistance of TiZrHfNb is superior to that of TiZrHfNbTa. Electrochemical tests show that TiZrHfNb has a more stable corrosion potential, lower passive current density, and more stable passive behavior in the case of chloride media. SEM and AFM analysis confirm that TiZrHfNb has an intact, smoother surface after immersion in AS with no indication of surface roughening or localized corrosion. This agrees with the formation of an unbroken passive film of stable mixed oxides of Zr, Nb, and Hf resistant to successful degradation on exposure to chlorides, irrespective of the presence or absence of Ta. However, TiZrHfNbTa demonstrates a strong corrosion resistance against fluoride-based ASF solution. Ta addition significantly enhances the alloy’s electrochemical stability in fluoride environment as indicated by a more noble corrosion potential, reduced passive current density, and greater passive region. The EIS analysis indicates the presence of a thin and protective passive layer with greater charge transfer and film resistance, supposedly due to Ta_2_O_5_-enrichment. Mott–Schottky analysis also reveals a reduced donor density for TiZrHfNbTa, consistent with a less defective and more protective oxide film. SEM and AFM surface analysis confirm that TiZrHfNbTa possesses a smoother, less degraded surface, with indications of nano-sized oxide particle growth consistent with passive film self-healing. These findings validate that TiZrHfNb is the most appropriate for exposures to chloride ions, and TiZrHfNbTa is the most appropriate for exposures to fluoride and illustrate the environment-dependent performance advantages of each alloy for long-term corrosion-resistant biomedical use.

## Figures and Tables

**Table 1:** Chemical composition of the HEA samples.

Element	Fractional Calculation					EDX Measurement	
	Molar Fraction (m)	Atomic Fraction (at. %)	Atomic Weight (Ar)	Ar × at. %	Weight Fraction (wt. %)	Atomic Fraction (at. %)	Weight Fraction (wt. %)
TiZrHfNb							
Ti	1	29.41	47.87	14.07	13.49	31.95	15.13
Zr	1	29.41	91.22	26.83	25.70	29.10	26.26
Hf	1	29.41	178.49	52.48	50.32	26.96	47.60
Nb	0.4	11.76	92.91	10.92	10.48	11.99	11.09
Total	3.4	100		104.3	100	100	100
TiZrHfNbTa							
Ti	1	20.00	47.87	9.57	8.09	21.89	8.88
Zr	1	20.00	91.22	18.24	15.42	17.89	13.91
Hf	1	20.00	178.49	35.70	30.17	17.83	21.75
Nb	1	20.00	92.91	18.58	15.71	20.64	16.34
Ta	1	20.00	180.95	36.19	30.61	21.89	33.75
Total	5	100		118.28	100	100	100

Note: at. % = (m/total m) × 100%; wt. % = (Ar × at. %)/total (Ar × at. %) × 100%.

**Table 2:** Chemical composition of AS solution.

Compound	NaCl	KCl	CaCl_2_·2H_2_O	Na_2_S·9H_2_O	NaH_2_PO_4_·2H_2_O	Urea
Concentration (g/L)	0.4	0.4	0.795	0.005	0.69	1

**Table 3:** Fitting parameters derived from the proposed equivalent circuit models.

Parameter	TiZrHfNb	TiZrHfNbTa	CP-Ti	Ti-6Al-4V	TiZrHfNb	TiZrHfNbTa	CP-Ti	Ti-6Al-4V
	AS Solution				ASF Solution			
*R_u_* (Ω)	25.10	25.69	24.35	25.50	34.37	35.75	35.24	36.00
*R*_*p*1_ (Ω)	3634.30	3269.50	2822.50	2500.70	1030.48	3569.90	2134.26	661.46
*Y*_01_ (×10^−6^ S·s^a^)	4.63	6.51	3.54	3.23	1.03	2.37	3.73	2.32
*a*_1_	0.96	0.83	0.94	0.81	0.86	0.87	0.86	0.84
*W_d_* (×10^−6^ S·s^½^)	1.23	12.3	17	19.3	–	199	–	–
*L* (H)	–	–	–	–	530.34	–	–	427.2
*R*_2_ (Ω)	–	–	–	–	576.53	–	–	576.54
*Y*_02_ (S·s^a^)	–	–	–	–	0.03	–	–	0.03
*a*_2_	–	–	–	–	–	–	–	0.82

**Table 4:** Corrosion parameters derived from the CPP results.

Parameter	TiZrHfNb	TiZrHfNbTa	CP-Ti	Ti-6Al-4V	TiZrHfNb	TiZrHfNbTa	CP-Ti	Ti-6Al-4V
	AS Solution				ASF Solution			
*i_corr_* (nA·cm^−2^)	26.98	18.38	84.20	71.91	61.92	40.70	74.36	101.04
*E_corr_* (mV)	147.70	300.10	275.2	289	−650.0	285.20	250.10	−663.40
*E_pass_* (V)	0.20	0.10	0.05	0.30	−0.20	0.30	0.10	−0.10
*E_pit_* (V)	2.00	1.80	1.20	1.60	1.10	1.40	1.20	0.50
*E_a/c_* (V)	−0.40	−0.30	−0.35	−0.25	0.80	0.90	0.80	0.70

**Table 5:** Mott–Schottky parameters derived from their plots.

Parameter	TiZrHfNb	TiZrHfNbTa	CP-Ti	Ti-6Al-4V	TiZrHfNb	TiZrHfNbTa	CP-Ti	Ti-6Al-4V
	AS Solution				ASF Solution			
Slope (×10^15^)	−0.43	−0.44	−0.02	1.37	−0.04	9.65	2.08	−0.23
*N_D_* (×10^20^ cm^−3^)	−2.6	−0.18	−4.7	81	−2.3	0.11	0.05	−4.8
Semiconductor type	P-type	P-type	P-type	N-type	P-type	N-type	N-type	N- to P-type

**Table 6:** Roughness parameters of HEA samples prior to and after PSP tests in AS and ASF solution.

Sample	Ra (nm)		
	Prior	AS Solution	ASF Solution
TiZrHfNb	0.61	1.53	106.77
TiZrHfNbTa	6.49	2.58	50.17

**Table 7:** EDX results for the corrosion products in the AS solution.

Element	TiZrHfNb		TiZrHfNbTa	
	At. %	Wt. %	At. %	Wt. %
O (K)	15.10	2.64	21.87	3.91
Ta (M)	-	-	10.16	20.57
Zr (L)	24.06	23.99	18.39	18.76
Nb (L)	10.23	10.39	12.78	13.28
Ti (K)	25.08	13.13	20.53	11.00
Hf (L)	25.54	49.84	16.27	32.47

**Table 8:** EDX results for the corrosion products in the ASF solution.

Element	TiZrHfNb		TiZrHfNbTa	
	At. %	Wt. %	At. %	At. %
C (K)	7.84	2.57	5.07	1.19
O (K)	38.28	16.73	7.27	2.27
F (K)	17.76	9.22	24.85	9.23
Na (K)	6.34	3.98	16.31	7.33
Hf (M)	5.41	26.39	7.46	26.03
Ta (M)	-	-	5.04	17.83
P (K)	3.68	3.11	12.23	7.41
Nb (L)	4.39	11.15	9.88	17.94
Cl (K)	0.45	0.44	0.57	0.39
Ca (K)	-	-	1.65	1.29
Ti (K)	10.56	13.81	9.69	9.07
Zr (L)	4.89	12.17	-	-
K (K)	0.4	0.42	-	-

## Data Availability

The original contributions presented in this study are included in the article. Further inquiries can be directed to the corresponding author.
